# Antimicrobial susceptibility, virulence determinants profiles and molecular characteristics of *Staphylococcus epidermidis* isolates in Wenzhou, eastern China

**DOI:** 10.1186/s12866-019-1523-6

**Published:** 2019-07-09

**Authors:** Yinjuan Guo, Yu Ding, Li Liu, Xiaofei Shen, Zhihao Hao, Jingjing Duan, Ye Jin, Zengqiang Chen, Fangyou Yu

**Affiliations:** 1 Department of Laboratory Medicine, Shanghai Pulmonary Hospital, Tongji University School of Medicine, Shanghai, 200082 China; 2 Department of Laboratory Medicine, Hunan Provincial People’s Hospital, Changsha, 410000 China; 3 Department of Laboratory Medicine, the First Affiliated Hospital of Wenzhou Medical University, Wenzhou, 325000 China

**Keywords:** *Staphylococcus epidermidis*, Resistance, Virulence-genes, MLST

## Abstract

**Background:**

*Staphylococcus epidermidis* has emerged as an often encountered pathogen responsible for hospital-acquired infections. The aim of present study is to investigate the microbiological characteristic of *S. epidermidis* isolates isolated from sterile specimens and skin in a Chinese tertiary hospital.

**Methods:**

A total of 223 non-duplicate *S. epidermidis* were collected from various sterile specimens of inpatients among 10 years in Wenzhou, China. 106 *S. epidermidis* obtained from the skin (urethral orifices) of healthy volunteers. All isolates were tested for antimicrobial susceptibility. PCR was used to detect the virulence- and resistance-associated genes and 7 housekeeping genes to determine the sequence types (STs) of selected isolates.

**Results:**

The resistance rates to antimicrobials tested except linezolid and vancomycin and the prevalence of methicillin-resistant *S. epidermidis* (MRSE) of *S. epidermidis* clinical isolates were significantly higher than those among colonized isolates (*P* < 0.05). The positive rates of virulence-associated genes including *aap*, *sesI*, ACME-*arcA*, *IS256*, *bhp, altE*, *aae* and *gehD* for *S. epidermidis* clinical isolates were significantly higher than those for colonized isolate (*P* < 0.05). A total of 60 STs including 28 from clinical isolates and 32 from colonized isolates were identified by MLST. A novel, rarely encountered clone, ST466, was found to be the second prevalent clone among clinical isolates. The great majority of the *S. epidermidis* isolates tested (73.86%) belonged to clone complex 2 (CC2). Compared with ST2, ST130, ST20 and ST59 clones, ST466 clone had the highest resistance rate to tetracycline (50.00%), the second highest prevalence of ACME-*arcA* (65.00%), *bhp* (30.00%) and *qacA/B* (65.00%), very low prevalence of carriage of *icaA* (0.00%) and biofilm formation (0.00%), the lack of *sesI* and high prevalence of *aap*, *altE* and *aae* (> 90%), which was similar to the characteristics of ST59 clone with one locus difference from ST466. ST466 clone competence with *Staphylococcus aureus* was relatively stronger, relative to ST2, ST20, ST130 and ST59 clones.

**Conclusion:**

Taken together, a high-level of genetic diversity was found between clinical and colonized *S. epidermidis* isolates. A novel ST466 clone with distinct and similar characteristics relative to other prevalent clones, emerging as a prevalent clone in China, should be of major concern.

**Electronic supplementary material:**

The online version of this article (10.1186/s12866-019-1523-6) contains supplementary material, which is available to authorized users.

## Background

*Staphylococcus epidermidis* is a common commensal bacterium, which belongs to coagulase-negative *Staphylococci* (CoNS). It is now recognized as a relevant opportunistic pathogen and isolated prevalently from human epithelia and colonizes predominantly the axillae, head and nares [1]. However, in recent decades, as the *S. epidermidis* is one of the often encountered biofilm-producing bacteria, it has emerged as a common cause of hospital-acquired infections associated with the use of indwelling or implanted foreign bodies [1]. Biofilm formation is the most important factor for the establishment of *S. epidermidis.* The principal component of biofilms a polysaccharide intercellular adhesin (PIA) or polymeric *N*-acetyl-glucosamine (PNAG) produced by the four gene *icaADBC* operon [2]. In addition to PIA, many components have been found to be associated with biofilm formation, such as accumulation-associated protein (Aap), biofilm-associated protein (Bap) and IS256. A novel genomic island named arginine catabolic mobile element (ACME) may increase the colonized capacity of *S. epidermidis* to the human skin, mucosal surfaces and in-dwelling medical devices [3]. Furthermore, as for nosocomial pathogen, increasing rates of antibiotic resistance are an even greater problem for *S. epidermidis* limits our therapeutic options. In previous decades, a continuous decreased susceptibility of *S. epidermidis* to most of the clinically available antibiotics was found. Methicillin resistance in *Staphylococci*, which is associated with the presence of the *mecA* gene carried by a genetic mobile element called the staphylococcal chromosomal cassette *mec* (SCC*mec*), is the other very important factor in the establishment of *S. epidermidis* as a nosocomial pathogen [1, 4]. Molecular typing has become establish for the population analysis of nosocomial *S. epidermidis* strain and is a good method for addressing the lack information compared with that of *S. aureus* [5]. By using multilocus sequence typing (MLST), a typing method based on the sequence polymorphism of fragments of seven housekeeping genes, it was revealed several epidemiology clonal lineages disseminated worldwide [5]. Clonal complex 2 (CC2), the most predominant of these lineages, is composed of a large number of sequence types [6, 7]. Limited information is available on the molecular epidemiology of *S. epidermidis* clinical isolates in China. In the present study, the aim is to investigate the antimicrobial resistance profiles, virulence determinants profiles and molecular characteristics of *S. epidermidis* isolates.

## Methods

### Bacterial isolates

- Clinical *S. epidermidis* isolates

A total of 223 non-duplicate *S. epidermidis* isolates were isolated from various sterile specimens of inpatients from 2002 to 2008 and 2012 to 2014 at the bacterial library of the first Affiliated Hospital of Wenzhou Medical University, Wenzhou, eastern China. Each isolates were transferred to a blood plate and then cultured overnight in 5 ml TSB at 37 °C with shaking at 200 rpm from glycerin broth stored in − 80 °C of bacterial library. The *S. epidermidis* isolates from patients with clinical signs and symptoms of infection were considered for invasive isolates and included for investigation. The isolates included were isolated from blood (67 isolates), catheters (52 isolates), pus (48 isolates, 7.9%) and other specimens (56 isolates).

b) Colonized *S. epidermidis* isolates

The definition of colonization is bacteria often fall from different environments to the human body, and can settle and grow in a certain part, and breed offspring. 106 colonized *S. epidermidis* isolates were isolated from the urethral orifices of healthy volunteers with no symptoms. There were 76% female and 24% male from 18 to 30 years old without any diseases. The isolates tested were identified as *S. epidermidis* by VITEK automatic microbiology analyzer (bioMe’rieux, Marcy l’Etoile, France).

### Antimicrobial susceptibility testing

In vitro antimicrobial susceptibility testing was determined by the disk diffusion method with antimicrobials in accordance with the standards recommended by the Clinical and Laboratory Standards Institute(CLSI) [8], including penicillin (10 μg), erythromycin (15 μg), clindamycin (2 μg), linezolid (30 μg), tetracycline (30 μg), trimethoprim/sulfamethoxazole (1.25/23.75 μg), gentamicin (10 μg), ciprofloxacin (5 μg), and levofloxacin (5 μg). All disks were obtained from Oxoid Ltd. *S. aueus* ATCC 25923 was used as control strain for determining the antimicrobial susceptibility for *S. epidermidis* isolates in accordance with CLSI breakpoints. The minimum inhibitory concentration value of vancomycin was determined with the standard agar dilution method recommended by the CLSI [8].

### MLST typing and goeBURST algorithm

Multilocus sequence typing of the *S. epidermidis* strains was determined by amplifying of the seven housekeeping genes including *arcC*, *aroE*, *gtr*, *mutS*, *pyrR*, *tpiA* and *yqiL* as described previously [9]. The fragments containing seven housekeeping genes were sent to TSINGKE Company (Hangzhou, China) for purified and DNA sequencing service. The numbers of alleles and sequence types were assigned using online database (http://www.mlst.net). The goeBURST algorithm (http://goeBURST.phyloviz.net) was used to infer the evolutionary relatedness of the STs.

### PCR method for detection of virulence- and resistance-associated genes

All isolates included were tested for the presence of the *mecA* gene by PCR amplification using the primers described previously [10]. The presence of the virulence-associated genes including *icaA*, *sesI*, *aap, arcA, IS256, altE, bhp, aae* and *gehD* was determined by PCR using primers described previously [7, 11–15]. PCR was used to detect whether the isolates tested harbored mupirocin-resistance protein-encoding gene (*mupA*) and chlorhexidine-based antiseptic resistance loci (*qacA/B*) according to previously described primers [16].

### Biofilm formation assay

The biofilm formation assay was performed as described by O’Neill et al. using the flat bottom 96-wellpolystyrene plates (BD Biosciences) [17]. In brief, each strain grew overnight in trypticase soy broth (TSB) (BD, USA). Then, the bacterial suspensions (1 × 10^8^ CFU/ml) were diluted 1:200 in fresh TSB. Wells of a 96-well microplate were then inoculated with two hundred microliter bacterial suspensions. TSB served as background controls. After 24 h incubation at 37 °C, the walls were removed and washed three times with 200 μl of PBS. Then the plates were stained with a 0.4% (w/v) crystal violet solution in 10 min and washed. Biofilm formation was decided by the 200 μl of 33% glacial acid for 10 min and measured the value of OD_492_ with a microplate reader. Each experiment was repeated three times separately. When the value of A_492_ of ≥0.17, we defined that it was a biofilm positive isolate. The *S. epidermidis* RP62A (ATCC 35984) and the *S. epidermidis* ATCC12228 were used as positive and negative controls, respectively.

#### Competition with *Staphylococcus aureus*

This assay was experimented according to the previous study [18]. Briefly, each tested *S. epidermidis* isolate grew overnight in TSB. After incubation at 37 °C, the turbidities of tested *S. epidermidis* isolates at 600 nm were measured using a microplate reader. The OD_600_ value (0.2) of each tested isolate was adjusted to the same concentration of 1.0 × 10^5^ CFU/100 μl. *S. aureus* 75 strain (SA75) associated with skin and soft tissue infection was selected as competence object of *S. epidermidis* isolates, and adjusted to the same concentration. Then SA75 and *S. epidermidis* isolates were simultaneously diluted to 1:200 and added into TSB liquid medium. After a 9 h incubation at 37 °C, the broth with mixture of *S. epidermidis* and *S. aureus* was diluted to 0.25 × 10^− 6^. 100 μl of the diluted culture was plated onto blood agar plates and incubated at 37 °C overnight. The amounts of colonies of *S. epidermidis* isolate and SA75 on the plate were counted, which was on the basis of SA75 isolate used being beta-haemolytic. The ratio of the numbers of colonies of tested *S. epidermidis* isolate and SA75 was used for evaluating the competence of *S. epidermidis* isolate with SA75. The experiment was repeated twice.

### Statistical analysis

Statistical software (version 19, IBM SPSS Statistics) was used for all data analyses.

Fisher’s exact test or the chi-square test was used to compare positive rates of virulence-associated genes and antimicrobial resistance. T-test was used to compare the competence among different STs. The competition assay were analyzed using Graphpad Prism software (version 7.00, La Jolla, CA, United States), and *p*-value< 0.05 was considered statistically significant. Statistical significance was achieved with 2-sided *P* value of 0.05.

## Results

### Antimicrobial susceptibility characteristics

97(43.49%) and 63 (59.43%) clinical and colonized isolates positive for *mecA* determined by PCR were identified as MRSE. The prevalence of MRSE among clinical isolates was significantly higher than that among colonized isolates (*P* < 0.01). The resistance rates of clinical and colonized isolates to antimicrobials tested were listed in Table 1. All tested isolates were susceptible to vancomycin. Only one colonized isolate were resistant to linezolid, while none of those clinical isolates were resistant to linezolid. The resistance rates of clinical isolates to penicillin, tetracycline, ciprofloxacin, chloramphenicol, gentamicin, clindamycin, erythromycin andtrimethoprim-sulfamethoxazole were significantly higher than those among colonized isolates.

**Table 1:** Antimicrobial resistance profiles and carriage of virulence genes among *S. epidermidis* clinical and colonized isolates

	Clinical isolates (*N* = 223)		Colonized isolates (*N* = 106)		*P* value
	No.	%	No.	%	
Antimicrobials					
penicillin	213	95.52	87	82.08	0.000
tetracycline	76	34.08	19	17.92	0.004
ciprofloxacin	66	29.60	37	34.91	0.000
chloramphenicol	78	34.98	10	9.43	0.000
gentamicin	72	32.29	21	19.81	0.000
clindamycin	110	49.33	36	33.96	0.000
erythromycin	184	82.51	66	62.26	0.000
Trimethoprim/sulfamethoxazole	99	44.39	27	25.47	0.000
linezolid	0	0.00	1	0.94	0.684
vancomycin	0	0.00	0	0.00	NA^a^
Biofilm formation	21	9.42	19	17.92	
Virulence genes					
*icaA*	46	20.63	24	22.64	0.202
*aap*	148	66.37	61	57.55	0.022
*sesI*	39	17.49	4	3.77	0.000
*arcA*	112	52.02	37	34.91	0.000
*IS256*	97	43.50	2	1.89	0.000
*Bhp*	102	22.32	0	0.00	0.000
*Aae*	443	96.94	13	12.26	0.000
*GehD*	168	36.76	7	6.60	0.000
Resistance genes					
*mupA*	41	18.38	11	10.38	0.065
*qacA/B*	100	44.84	57	53.77	0.058

^a^ *NA* not analyzed

### Prevalence of *mupA* and *qacA/B*

The positive rates of *mupA* and *qacA/B* among clinical and colonized *S. epidermidis* isolates were 18.38 and 44.84%, and 10.38 and 53.77%, respectively. Although the prevalence of *mupA* among clinical isolates was higher than that of colonized isolates, there was no difference between two groups. But the prevalence of *qacA/B* was reserved.

### Prevalence of virulence-associated genes and biofilm formation

The positive rates of virulence-associated genes among clinical and colonized *S. epidermidis* isolates were showed in Table 1. *IS256* was found among 43.49% of clinical isolates while only 1.89% of colonized isolates. In the present study, ACME-*acrA* was also found both in clinical (52.02%) and colonized (34.91%) *S. epidermidis* isolates. The positive rates of *altE* and *aae* among clinical isolates were high to 97.76%, while only 13.21 and 12.26% among colonized isolates. The positive rates of virulence-associated genes except *icaA* among *S. epidermidis* clinical isolates were significantly higher than those among colonized isolates(*P* < 0.05).There were no differences in the positive rates of biofilm formation between clinical isolate and colonized isolates. However, the positive rates of another biofilm-associated gene, *aap*, among clinical and colonized isolates were relatively high.

### Genetic diversity of *S. epidermidis* clinical and colonized isolates

**Fig. 1:**
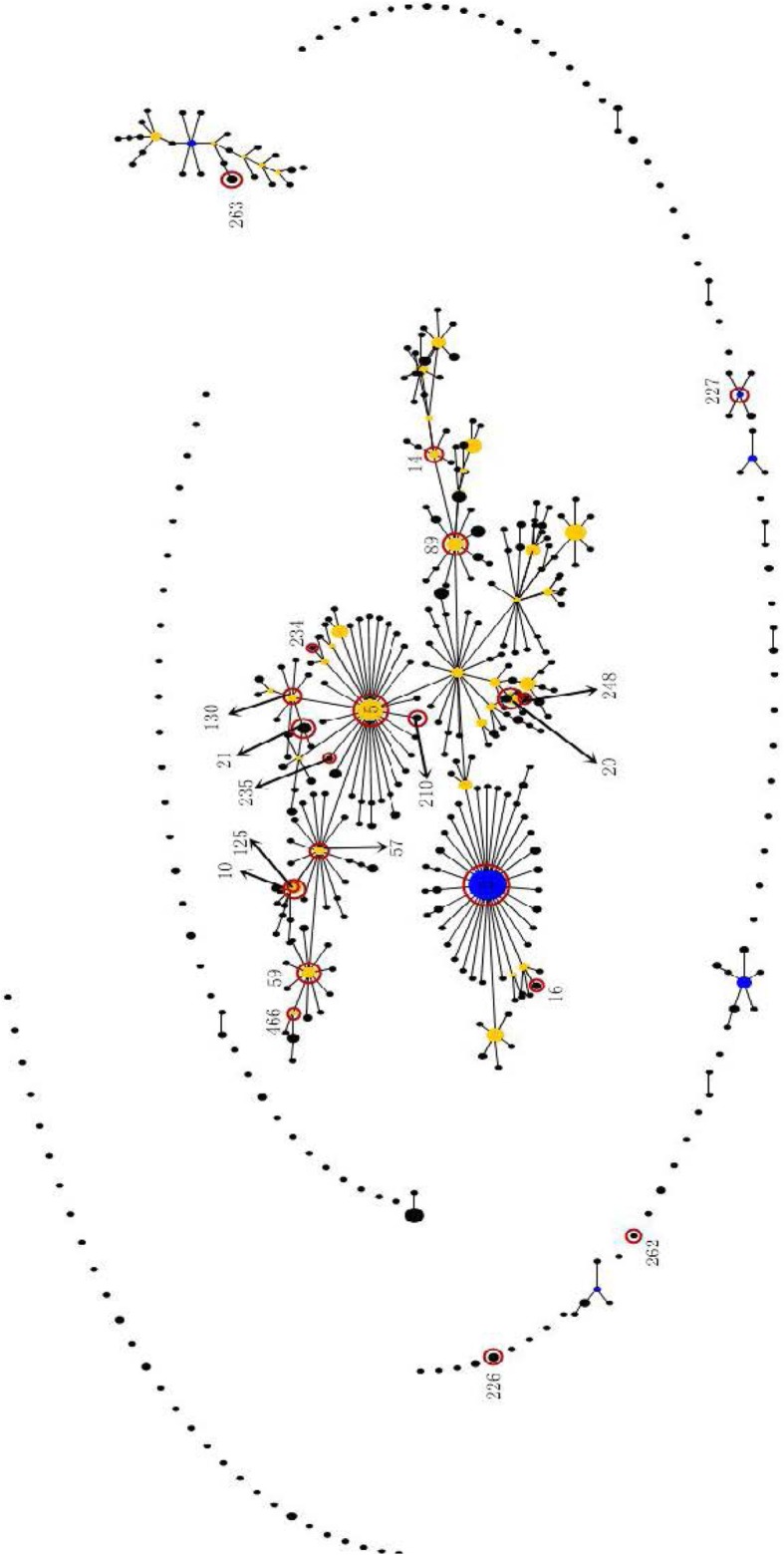
eBURST analysis of *S.epidermidis* using all STs available in the MLST database. ST2 was the founder of *S. epidermidis* in MLST database. ST5, the single locus variant (SLV) of ST2, was the founder of clinical isolates, while ST85, also the SLV of ST2, was the founder of colonized isolates

A total of 60 STs were identified by MLST among the 329 isolates analyzed (Table 2). The Fig. 1 were showed that eBURST analysis of *S.epidermidis* using all STs available in the MLST database. Among the 223 clinical isolates, 28 STs were identified, with ST2 the most prevalent ST (28 isolates, 12.56%), followed by ST466 (20 isolates, 8.97%). The other STs were included merely one or several strains. 32 STs were identified among 106 colonized isolates. The major STs were ST130 (19 isolates, 17.92%), ST466 (16 isolates, 15.09%) and ST20 (14 isolates, 13.21%). However, only one colonized isolate belonged to ST2. The other 29 dispersed STs correspond to 53.77% of the colonized isolates. Moreover, the STs of 13 clinical and 12 colonized isolates were not matched with known STs in online database (http://www.mlst.net), which may be novel STs. Twenty one STs listed in Table 2 were exclusively found among clinical isolates. Six STs including ST32, ST83, ST138, ST141, ST230 and ST325 were exclusively identified among colonized isolates. Twenty-seven STs mainly including ST466, ST130, ST20, ST59 and ST14 were found both clinical and colonized isolates. The prevalent STs accounting for more than 20 clinical isolates, including ST2, ST466, ST59, ST130 and ST20 were designated as major clones.

**Table 2:** Sequence types (ST) distribution among *S. epidermidis* clinical isolates and colonized isolates

MLST	CC	Clinical isolates(*N* = 223)		Colonized isolates(*N* = 106)	
		No.	%	No.	%
ST2	2	28	12.56%	1	0.94%
ST5	2	1	0.45%	0	0.00%
ST10	2	2	0.90%	0	0.00%
ST14	2	7	3.14%	5	4.72%
ST16	2	2	0.90%	0	0.00%
ST20	2	8	3.59%	14	13.21%
ST21	2	3	1.35%	0	0.00%
ST57	2	4	1.79%	2	1.89%
ST59	2	12	5.38%	3	2.83%
ST89	2	3	1.35%	0	0.00%
ST125	2	6	2.69%	1	0.94%
ST130	2	16	7.17%	19	17.92%
ST210	2	9	4.04%	1	0.94%
ST226	Singletons	4	1.79%	0	0.00%
ST227	227	5	2.24%	1	0.94%
ST234	2	3	1.35%	1	0.94%
ST235	2	6	2.69%	0	0.00%
ST248	2	1	0.45%	1	0.94%
ST262	Singletons	4	1.79%	0	0.00%
ST263	365	5	2.24%	1	0.94%
ST466	2	20	8.97%	16	15.09%
Non typing		13	5.83%	12	11.32%
Other		61	27.35%	28	26.42%
Total		223	100.00%	106	100.00%

*CC* clonal complex

**Fig. 2:**
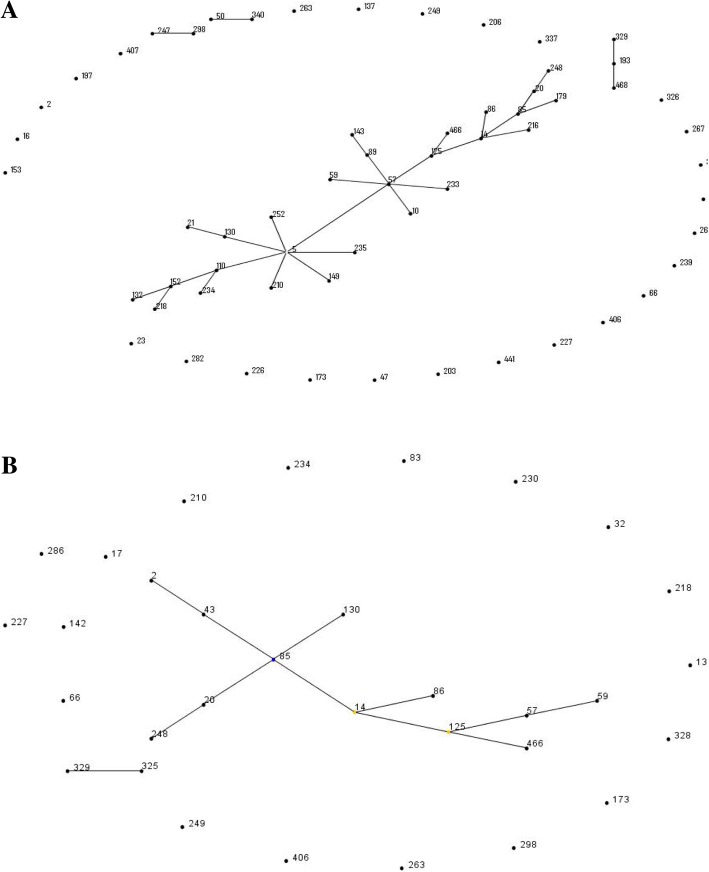
eBURST analysis of *S. epidermidis* clinical isolates (**a**) and colonized isolates (**b**). The eBURST algorithm clustered all of the 60 STs isolated of clinical isolates into clonal complex 2 (CC2) (166/223; 74.40%), 7 minor CCs (CC365, CC193, CC227, CC171, CC33, CC326, and CC66, 16/223, 7.17%), and 29 singletons. Among the 106 colonized isolates, 5 CCs were identified, including CC2 (77/106; 72.64%), CC365 (4 isolates), CC193 (1 isolates), CC227 (1 isolates) and CC66 (1 isolates)

The eBURST algorithm clustered all of the 60 STs isolated of clinical isolates into clonal complex 2 (CC2) (166/223; 74.40%), 7 minor CCs (CC365, CC193, CC227, CC171, CC33, CC326, and CC66, 16/223, 7.17%), and 29 singletons. Among the 106 colonized isolates, 5 CCs were identified, including CC2 (77/106; 72.64%), CC365 (4 isolates), CC193 (1 isolates), CC227 (1 isolates) and CC66 (1 isolates) as shown in Fig. 2b. ST2 was the founder of *S. epidermidis* in MLST database. In the present study, ST5, the single locus variant (SLV) of ST2, was the founder of clinical isolates, while ST85, also the SLV of ST2, was the founder of colonized isolates. Although 60 different STs were identified among *S. epidermidis* isolates analyzed, the great majority of the isolates (73.8%, 243/329) belonged to a single clone complex, CC2.

### Comparison of characteristics of ST466 clone with other major clones among clinical isolates

The characteristics of 5 major clones were showed in Table 3.

- Antimicrobial resistance profiles and prevalence of *mupA* and *qacA/B*

**Table 3:** The antimicrobial resistance rates, positive rates of virulence genes and of resistance genes among *S.epidermidis* clinical isolates with major ST types (%)

	ST466 (*N* = 20)	ST2 (*N* = 28)	ST130 (*N* = 16)	ST20 (*N* = 8)	ST59 (*N* = 12)
Antimicrobials					
penicillin	95(19)	96.43(27)	100.00(16)	100.00(8)	91(11)
tetracycline	50.00(10)	10.71(3)	25.00(4)	50.00(4)	33.33(4)
ciprofloxacin	15.00(3)	82.14(23)	25.00(4)	50.00(4)	33.33(4)
chloramphenicol	30.00(6)	25.00(7)	12.50(2)	12.50(1)	0.00(0)
gentamicin	20.00(4)	82.14(23)	25.00(4)	0.00(0)	33.33(4)
clindamycin	30.00(6)	67.86(19)	50.00(8)	25.00(2)	41.67(5)
erythromycin	80.00(16)	82.14(23)	68.75(11)	87.50(7)	83.33(10)
SXT^a^	60.00(12)	71.43(20)	100(16)	0.00(0)	41.67(5)
linezolid	0.00(0)	0.00(0)	0.00(0)	0.00(0)	0.00(0)
vacomycin	0.00(0)	0.00(0)	0.00(0)	0.00(0)	0.00(0)
Biofilm formation	5.00(1)	10.71(3)	0.00(0)	75.00(6)	0.00(0)
Virulence genes					
*icaA*	0.00(0)	85.71(24)	0.00(0)	62.50(5)	0.00(0)
*aap*	90.00(18)	96.43(27)	50.00(8)	50.00(8)	91.67(11)
*sesI*	0.00(0)	96.43(27)	0.00(0)	0.00(0)	25.00(3)
ACME-*arcA*	65.00(13)	39.29(11)	87.50(14)	25.00(2)	41.67(5)
*IS256*	15.00(3)	82.14(23)	43.75(7)	25.00(2)	33.33(4)
*bhp*	30.00(6)	10.71(3)	0.00(0)	0.00(0)	50.00(6)
*aae*	100.00(20)	92.86(26)	100.00(16)	100.00(8)	100.00(12)
*altE*	100.00(20)	96.43(27)	100.00(16)	100.00(8)	100.00(12)
*gehD*	25.00(5)	67.86(19)	12.50(2)	62.50(5)	33.33(4)
Resistance genes					
*mupA*	0.00(0)	39.29(11)	12.50(2)	12.50(1)	8.33(1)
*qacA/B*	65.00(13)	67.86(19)	56.25(9)	62.50(5)	58.33(7)

^a^ *SXT* trimethoprim/sulfamethoxazole

Interestingly, the prevalence of MRSE among these 5 major clones was high to more than 95%, especially ST59 and ST20 clones with MRSE prevalence of 100%. The resistance rates of ST2 clone to ciprofloxacin, gentamicin, clindamycin, erythromycin and trimethoprim-sulfamethoxazole were more than 65%, which were higher than other clones, but with the lowest resistance rate of tetracycline(10.71%). In contrast to ST2 clone, the second prevalent clone, ST466, had the highest resistance rate to tetracycline (50.00%) relative to other clones. The resistance rates of ST466 clone to antimicrobials tested were similar to ST59 clone except chloramphenicol. The resistance rates of ST20 clone to trimethoprim-sulfamethoxazole, gentamicin, clindamycin and chloramphenical were lower than other clones, especially to trimethoprim-sulfamethoxazole(0.0%). The positive rates of *mupA* and *qacA/B* among ST2 clone were higher than other 4 clones. The prevalence of *qacA/B* among ST466 clone was near to that of ST2 and 59 clones, with a relatively high prevalence of *qacA/B*.

b) Biofilm formation and carriage of virulence-associated genes

Although the prevalence of biofilm formation among clinical isolates was only 9.42%, that among ST20 clone was high to 75.00%, followed by ST2 clone (10.71%), which was associated with more than 60% of ST2 and ST20 clones with *icaA*. Conversely, among clinical isolates all ST466 clone except one isolate and ST59 clone were negative for *icaA* and biofilm formation. In the present study, the prevalence of *IS256* among ST2 clone was 82.14%, which was significantly higher than other clones. The highest prevalence of colonization-associated element, ACME, was found among ST130 clone (87.50, 14/16), followed by ST466 clone (65.00%, 13/20).The prevalence of *sesI* among ST2 clone was high to 96.43% (27/28). However, the positive rates of *sesI* among other 4 clones were low, especially no ST466 clone positive for this gene. Other than ST130 clone with relatively low *aap* prevalence (50.00%, 8/16), the positive rates of *aap* for ST2 (96.43%, 27/28), ST466 (90.00%, 18/20), ST59 (91.67%, 11/12) and ST20 (100.00%, 8/8) clones were over 90%. The positive rates of *altE* and *aae* among 5 prevalent clones were high to more than 90%, with all ST20, ST130 and ST59 clones positive for these two surface-associated autolysin/adhesion genes. The pattern of virulence-associated genes among ST466 was similar to ST59 except *sesI*. Compared with the predominant ST2 clone, ST466 clone had higher positive rates of ACME-*arcA*, *altE*, *aae* and *bhp*.

c) Competence with *S. aureus* of ST466

**Fig. 3:**
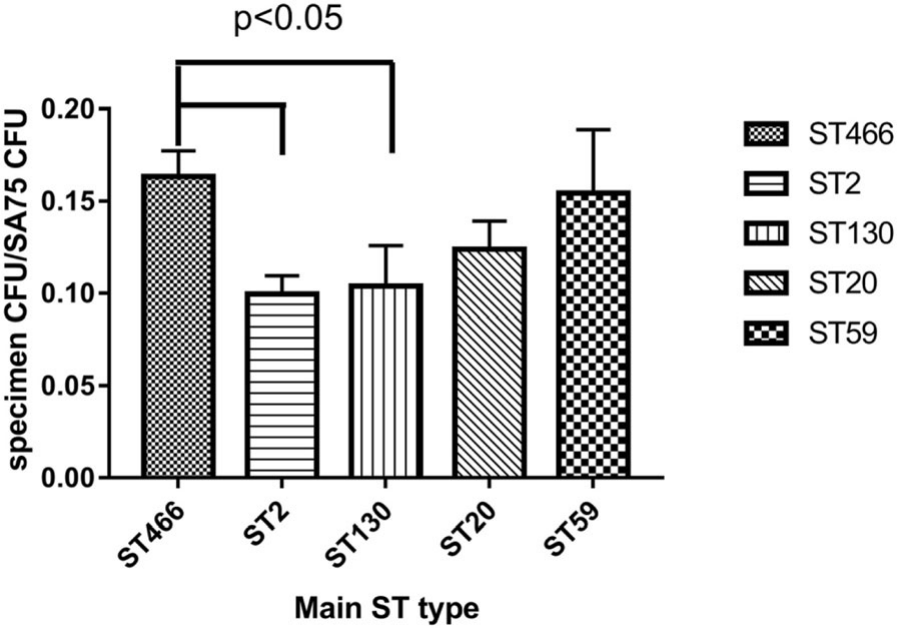
The competence of ST466 clone with *S. aureus* (SA75) were significantly stronger than ST2 and ST130 clones (Fig. 3) (*P* < 0.05). Although the competence of ST466 clone with *S. aureus* was higher than ST20 and ST59 clones, there were no differences among them, especially ST59 with similar competence relative to ST466

The competence of ST466 clone with *S. aureus* were significantly stronger than ST2 and ST130 clones (Fig. 3) (*P* < 0.05).Although the competence of ST466 clone with *S. aureus* was higher than ST20 and ST59 clones, there were no differences among them, especially ST59 with similar competence relative to ST466.

## Discussion

Both similarities and differences were detected in the genotypes, virulence-associated genes, antimicrobial susceptibility characteristics, ability of biofilm formation, STs and CC amongst *S.epidermidis* from clinical isolates and colonized isolates. Previous study showed that *S. epidermidis* isolates from patients with catheter-related bloodstream infection were significantly more resistant to most of non-beta lactam drugs than the *S. epidermidis* isolates collected from healthcare workers [19]. However, the resistance rates to clindamycin among *S. epidermidis* isolates were different from country to country. In most countries, clindamycin resistance rates were relatively high (> 40%) [20–24]. The resistance rates of *S. epidermidis* clinical isolates to clindamycin in Northern of Jordan (57.4%) [25] and Belgian (75%) [19] were relatively high. In contrast, a previous report from UK showed that resistance rate of *S. epidermidis* isolates from10 hospitals to clindamycin was low to 13% (82/633) [26].

Surface-active antiseptics such as chlorhexidine and mupirocin treatment were comprehensive strategy in reducing *S. aureus* and *S. epidermidis* colonization and infection in the hospital were strongly recommended as decolonization agents. Emergence of resistance to antiseptics and mupirocin will threaten the hospital infection control. In the present study, a high prevalence of *mupA* and *qacA/B* was found among *S. epidermidis* clinical and colonized isolates, which should be of concern.

Numerous infections caused by *S. epidermidis* involves biofilm formation, which is the most important factor involved in its pathogenesis [27].Some studies have shown that commensal *S. epidermidis* populations differ from clinical isolates in the frequency of the carriage of virulence genes such as *icaA* involved in biofilm formation [28–32]. Although no virulence determinants can be used to clearly distinguish *S. epidermidis* clinical isolates from colonized isolates [33, 34], *IS256* was found different between clinical isolates and colonized isolates. Previous study indicated that *sesI* may be a marker of *S. epidermidis* invasive capacity, because it was not found among healthy individuals, but was found in clinical isolates (approximately 50%) associated invasive infections [12]. In the present study, although the *sesI* prevalence in clinical isolates was lower than previous studies [12, 35], it was in accordance with the findings that *sesI* is more frequently found among clinical isolates than that in colonized isolates. A novel genomic island named arginine catabolic mobile element (ACME) may increase the colonized capacity of *S. epidermidis* to the human skin, mucosal surfaces and in-dwelling medical devices [36]. ACME was found in both clinical and colonized *S. epidermidis* isolates, indicating that ACME played a more important role in colonization than in virulence [37]. In the present study, the percentage of ACME-*acrA* was lower than previous reports (> 70%) [7, 37], but similar to the previous study that indicating the percentage of ACME-*acrA* of *S. epidermidis* isolates from a widespread geographical origin was 51% [38].

A surface-associated autolysin/adhesin, AtlE, from *S. epidermidis* mediates initial adherence of bacterial cells to the polymer surface [39]. Interestingly, all the isolates harboring *altE* except one colonized isolate were simultaneously positive for *aae* encoding another multifunctional autolysin/adhesin with bacteriolytic activity that binds to fibrinogen, vitronectin, and fibronectin. Bhp, a protein homologous to Bap was assumed to promote biofilm formation [1]. Surprisingly, *bhp* was exclusively found among clinical isolates, indicating that there was an association between carriage of *bhp* and pathogenicity of *S. epidermidis*.

Different results of ST type distribution indicated that the *S. epidermidis* tested in the present study showed considerable genetic diversity. The comparison of the isolated from the patients and healthy volunteers showed that two groups had a high level of genetic diversity, but the clinical isolates were even more diverse than the colonized isolates. Characteristics of highly diversity of *S. epidermidis* isolates were also observed in other studies [29, 40]. It was speculated that this genetic diversity might be caused by the need of the *S. epidermidis* to adapt to the different environments in the hospital and community settings [6]. ST2 has usually been the most prevalent ST observed in previous epidemiological studies among *S.epidermidis* strains worldwide, especially in BSIs and catheter-related infections [6, 41]. Furthermore, ST2 has been associated with many of the currently known virulence factors, such as biofilm production and antibiotic resistance [7, 41]. In the present study, ST2 was the most dominant type in clinical isolates, which was similar to previous studies [6, 41–44]. Interestingly, ST466 was the second prevalent ST among clinical isolates and colonized isolates. Up to now, only a report from Shanghai of China found that a minority of *S. epidermidis* nasal isolates from healthcare staff at a teaching hospital belonged to ST466 [7]. In addition to China, other countries did not found ST466. The spread of ST466 clone between clinical and colonized isolates indicated that this clone may be become the main cause of infections by *S. epidermidis* in China, which should be of concern.

In our study, the high prevalence of CC2 has been found both in clinical isolates and colonized isolates, indicating that CC2 possibly adapted to different environment by recombination or mutation. A report from China showed that 91.7% (297/ 324) of *S. epidermidis* from the community and hospital environments belonged to CC2 [7]. Our data support the evidence that overwhelming majority of *S. epidermidis* isolates from China belong to CC2 regardless of clinical isolates or colonized isolates. CC2 comprised of 74% of the *S. epidermidis* isolates from 17 national centers between 1996 and 2001 [6]. The majority (62/71, 87.3%) of *S. epidermidis* clinical isolates from U.S. hospitals belonged to CC2 [45]. Although molecular typing of *S. epidermidis* isolates from diverse geographic or clinical origins by MLST has shown considerable diversity, as shown in Fig. 2,CC2 is an overwhelming clonal lineage worldwide.

Our study showed that different clones were associated with characteristic non-β-lactam antimicrobial resistance and resistance genes patterns, suggesting that the clinical selection of antimicrobials based on typing molecular is help for the treatment of *S. epidermidis* infections and decolonization of *S. epidermidis*. Our data also suggested that high prevalence of MRSE, multiple antibiotic resistance and genes responsible for resistance to surface-active antiseptics and mupirocin used for decolonization of *Staphylococci*, contributes to the spread of the prevalent *S. epidermidis* clones in hospital environment, including a novel and rarely encountered clone, ST466.

A report from China showed that majority of the predominant clinical MRSE clone ST2 isolates were positive for biofilm-related genes *IS256* (81.0%) and *icaA* (75.0%) [7]. The high prevalence of virulence-associated genes and resistance to antimicrobials in ST2 of *S. epidermidis* isolates indicating that ST2 were associated with spread. In the present study, majority of *sesI*-positive *S. epidermidis* clinical isolates belonged to ST2, while few *sesI*-negative clinical isolates belonged to this clone, indicating that there was strong association between carriage of *sesI* and ST2 clone. While percentage of *sesI* and *icaA* in ST466 was 0.0%, we speculated that the prevalence of ST466 may not depend on formation of biofilm.The carriage of high prevalence of multiple virulence-associated genes including ACME-*arcA*, *altE*, *aae, aap* and *bhp* maybe contribute to the spread of ST466 clone. As ST466 was a single locus variant of ST59, the antimicrobial resistance profiles and the patterns of resistance genes and virulence-associated genes for ST466 clone were similar to those for ST59 clone.

As cross-interfering quorum-sensing pheromones were discovered in *staphylococci*, quorum-sensing cross-inhibition was believe to be the source for a potential interference between *S. epidermidis* and *S. aureus* [34]. Colonization with *S. epidermidis* can prevent overgrowth of the more aggressive *S. aureus* [46]. *S. epidermidis* strains were reported by Iwase et al. to can express a certain secreted protease Esp which prevented nasal colonization with *S. aureus* [47]. The underlying mechanism that ST466 clone had more competence with *S. aureus* relative to other 4 major clones was not known and should be further investigated.

## Conclusions

Taken together, a high-level of genetic diversity was found between clinical and colonized *S. epidermidis* isolates, with clinical *S. epidermidis* isolates with more resistance to clinically often used antimicrobial agents and carriage of virulence-associated genes. A novel ST466 clone with distinct and similar characteristics relative to other prevalent clones, emerging as a prevalent clone in China, should be of major concern.

## Additional files

Additional file 1: Drug susceptibility results of 223 strains of clinical *S. epidermidis*”. MIC values of 10 drug susceptibility results of 223 clinical *S. epidermidis*. (PDF 194 kb)

Additional file 2: Drug susceptibility results of 106 strains of colonized *S. epidermidis*”. MIC values of 10 drug susceptibility results of 106 colonized *S. epidermidis*. (PDF 112 kb)

## Data Availability

The datasets used during the current study are available from the corresponding author upon reasonable request. Most of the data is included in this published article [and Additional files 1 and 2].

## Abbreviations

ACME: Arginine catabolic mobile element
CC2: Clone complex 2
CLSI: Clinical and Laboratory Standards Institute
CoNS: Coagulase-negative *Staphylococci*
MLST: Multi-locus sequence typing
MRSE: Methicillin-resistant *S. epidermidis*
PIA: Polysaccharideinter cellular adhesion
PNAG: Polymericn-acetyl-glucosamine
SCC*mec*: Staphylococcal chromosomal cassette *mec*
