# Supplementary material for: Antimicrobial susceptibility, virulence determinants profiles and molecular characteristics of Staphylococcus epidermidis isolates in Wenzhou, eastern China
Source: BMC Microbiol. 2019 Jul 9;19:157. doi: 10.1186/s12866-019-1523-6 (PMC6617921; doi:10.1186/s12866-019-1523-6)
Supplement: Supplementary file 1 — Drug susceptibility results of 223 strains of clinical S. epidermidis”. MIC values of 10 drug susceptibility results of 223 clinical S. epidermidis. (PDF 194 kb) [file 12866_2019_1523_MOESM1_ESM.pdf]

No.	TE		SXT		CN		P		DA		E		CIP		FOX		C		LZD		VA(MIC)	
1	S	22	S	18	S	18	R	6	I	16	S	26	S	24	S	28	S	20	S	22	S	0.5
2	R	14	S	18	S	20	R	18	S	28	R	10	S	24	R	16	S	20	S	28	S	1
3	R	6	R	6	R	12	R	15	R	10	R	10	R	14	R	6	I	15	S	24	S	0.5
4	R	6	S	18	R	6	R	6	R	12	R	8	S	22	R	6	R	10	S	32	S	1
5	R	6	S	20	R	6	R	6	S	26	R	8	S	24	R	6	S	19	S	29	S	0.3
6	R	6	S	22	S	18	R	6	S	30	R	10	S	24	R	6	I	15	S	30	S	1
7	S	20	R	6	S	18	R	12	S	28	S	30	S	28	R	6	R	6	S	24	S	0.3
8	R	12	R	6	I	13	R	16	R	12	R	12	I	18	R	14	S	22	S	26	S	0.5
9	I	16	R	6	R	6	R	16	R	12	R	12	S	24	R	6	R	8	S	26	S	1
10	R	6	S	22	S	20	R	16	S	28	S	26	S	22	R	6	S	20	S	22	S	0.5
11	R	6	S	22	S	20	R	16	S	26	R	10	S	24	R	22	S	20	S	28	S	0.5
12	S	28	S	22	R	6	R	6	S	26	R	6	S	24	S	32	S	22	S	24	S	0.3
13	S	28	R	6	R	6	R	18	R	6	R	6	I	18	R	6	R	8	S	30	S	0.5
14	S	20	R	6	S	18	R	6	R	6	R	6	R	6	R	6	R	6	S	26	S	0.5
15	R	12	R	6	R	6	R	6	R	6	R	6	I	17	R	14	R	6	S	26	S	1
16	R	6	R	6	S	20	R	12	S	28	R	10	I	16	R	6	R	6	S	26	S	0.5
17	R	6	R	6	R	6	R	20	R	14	R	10	I	18	R	6	R	6	S	30	S	0.5
18	R	6	R	6	R	6	R	20	R	8	R	10	S	24	R	6	R	10	S	30	S	1
19	S	28	R	10	S	18	R	18	R	6	R	8	R	12	R	12	R	10	S	29	S	0.5
20	S	28	R	6	R	8	R	18	R	6	R	8	I	18	R	6	S	20	S	28	S	1
21	S	26	S	20	S	18	S	34	S	24	S	30	I	18	R	6	R	8	S	24	S	1
22	R	6	S	20	S	18	R	6	S	30	R	8	I	18	R	22	R	8	S	30	S	1
23	R	6	R	10	S	20	R	18	S	26	R	8	S	26	S	30	S	20	S	30	S	1
24	S	22	R	10	R	10	R	18	S	26	S	28	S	26	S	30	S	22	S	28	S	0.3
25	R	10	I	14	S	16	R	6	S	26	R	12	I	20	R	6	R	8	S	28	S	0.5
26	I	16	S	22	S	16	R	20	S	30	R	10	S	24	R	6	S	20	S	22	S	0.5
27	S	26	R	6	R	10	R	20	R	6	R	6	R	6	R	6	S	20	S	28	S	0.5
28	S	26	S	22	S	18	S	34	S	28	S	30	S	26	S	32	S	20	S	26	S	1
29	R	6	I	14	S	18	R	6	S	28	R	6	I	20	R	6	R	6	S	29	S	1
30	R	6	R	6	I	14	R	18	R	6	R	6	R	6	R	24	S	20	S	24	S	0.5
31	S	28	R	6	S	20	R	18	S	28	S	29	S	28	R	6	S	20	S	24	S	0.5
32	S	28	S	18	S	20	R	20	R	6	R	6	I	20	R	6	S	22	S	4	S	1
33	R	6	R	6	S	20	R	20	S	26	R	6	S	26	R	6	S	22	S	24	S	1
34	S	26	S	18	S	18	R	20	S	26	R	12	I	18	R	10	S	20	S	32	S	2
35	R	6	R	6	S	18	R	18	S	30	R	8	S	26	S	34	S	20	S	32	S	1
36	S	26	S	20	R	8	R	18	S	30	R	8	S	24	R	6	S	20	S	30	S	1
37	S	22	R	6	R	8	R	22	R	6	R	8	S	24	R	6	R	8	S	30	S	0.3
38	R	12	S	20	R	8	R	22	S	25	R	8	I	18	R	6	R	8	S	28	S	0.5
39	R	12	R	8	R	6	R	24	R	6	R	6	I	16	R	6	S	20	S	28	S	0.5
40	S	28	R	8	S	18	R	18	R	12	R	6	I	16	R	22	R	6	S	22	S	1
41	S	22	S	20	R	6	R	24	R	14	R	6	R	6	R	16	S	19	S	28	S	0.5
42	S	28	I	12	S	20	R	18	R	6	R	6	R	6	R	6	R	10	S	28	S	2
43	S	28	S	20	S	20	R	24	S	27	R	6	S	27	R	6	S	22	S	30	S	1
44	R	6	S	20	I	14	R	18	S	24	S	29	S	24	R	6	S	20	S	30	S	1
45	R	6	S	18	I	14	R	26	S	26	R	10	S	30	S	30	S	20	S	24	S	1
46	R	6	S	18	S	18	R	26	S	26	R	12	I	16	R	6	S	19	S	26	S	0.3
47	S	22	S	18	S	18	R	24	S	30	R	12	S	28	R	6	S	20	S	26	S	1
48	R	6	R	6	S	16	R	24	S	28	S	30	S	28	R	24	S	18	S	22	S	1
49	R	12	R	10	S	16	R	22	S	30	R	12	S	24	R	6	S	20	S	28	S	2

50	S	28	S	16	S	16	R	19	S	26	R	12	S	24	S	30	S	20	S	26	S	0.5
51	R	6	R	6	R	6	R	6	S	26	S	20	R	14	R	6	R	6	S	26	S	0.5
52	S	26	S	20	S	20	R	18	S	26	S	20	R	14	R	6	S	22	S	32	S	0.5
53	S	26	S	20	R	6	R	18	R	12	R	6	R	14	R	6	S	24	S	32	S	0.5
54	S	22	R	6	R	6	R	18	R	6	R	6	R	6	R	6	S	20	S	30	S	1
55	S	28	S	20	S	18	R	18	R	6	R	6	S	28	R	6	S	20	S	30	S	1
56	S	28	R	6	R	6	R	6	R	6	R	6	R	12	R	6	S	20	S	22	S	1
57	I	18	R	6	S	18	R	18	R	10	R	6	R	12	R	6	R	6	S	26	S	0.5
58	S	26	R	6	R	6	R	18	R	6	R	10	R	6	R	6	S	18	S	24	S	0.5
59	R	6	S	22	S	20	R	18	S	26	R	10	S	26	R	16	S	20	S	26	S	1
60	R	6	S	18	S	20	R	6	S	26	S	30	R	8	R	12	S	22	S	28	S	0.5
61	R	12	R	6	S	20	R	18	R	6	R	12	R	8	R	6	R	6	S	28	S	1
62	S	28	R	6	R	6	R	18	R	6	R	12	R	6	R	6	S	19	S	28	S	1
63	S	26	R	6	R	6	R	20	S	30	S	29	R	6	R	6	R	6	S	24	S	0.5
64	S	26	R	6	I	13	R	20	I	18	S	28	S	26	R	22	S	18	S	24	S	1
65	R	6	S	18	S	16	R	20	S	26	R	6	I	18	R	6	S	20	S	22	S	0.5
66	S	22	S	18	S	16	S	34	S	26	S	28	S	26	S	32	S	22	S	24	S	0.5
67	S	20	R	6	R	6	R	6	R	6	R	12	R	8	R	6	R	10	S	24	S	1
68	S	28	R	8	R	6	R	20	R	6	R	12	R	8	R	6	R	6	S	30	S	0.5
69	S	26	R	8	R	6	R	20	R	6	R	12	S	26	R	6	R	6	S	30	S	0.5
70	S	22	R	8	R	10	R	20	S	28	S	29	R	12	R	14	S	22	S	30	S	2
71	R	6	R	8	R	10	R	26	R	14	R	6	S	26	R	18	R	6	S	30	S	2
72	S	28	R	8	R	10	R	16	R	6	R	6	I	16	R	6	R	6	S	22	S	1
73	R	6	R	10	R	6	R	18	R	6	R	6	I	16	R	6	R	6	S	30	S	0.3
74	S	22	R	6	S	20	R	20	R	8	R	12	R	12	R	6	S	20	S	24	S	0.5
75	R	14	S	20	R	6	R	20	I	16	R	12	I	16	R	6	R	6	S	30	S	1
76	S	22	S	20	S	20	R	22	S	26	S	28	S	26	S	30	R	6	S	24	S	0.5
77	R	12	R	6	R	6	R	22	R	6	R	12	R	6	R	22	S	20	S	24	S	1
78	S	26	R	6	R	8	R	6	R	6	R	12	R	6	R	6	R	8	S	24	S	0.3
79	S	26	R	6	R	8	R	22	I	16	R	12	I	16	R	6	R	8	S	22	S	1
80	S	26	I	12	S	16	R	22	I	16	R	12	S	26	R	6	I	16	S	27	S	1
81	R	6	R	6	S	16	R	22	I	18	R	6	S	26	R	22	S	22	S	24	S	0.5
82	R	6	S	20	S	18	R	18	R	6	R	6	R	12	R	6	S	20	S	25	S	0.5
83	R	6	R	6	I	14	R	22	R	6	R	6	I	18	R	6	S	20	S	30	S	0.5
84	S	20	S	20	S	18	R	6	R	6	R	6	I	18	R	16	R	6	S	30	S	1
85	R	6	R	6	R	6	R	6	R	6	R	12	S	28	R	6	S	20	S	30	S	0.5
86	S	26	S	20	I	14	R	16	S	26	S	28	S	26	R	6	S	18	S	30	S	1
87	S	28	S	20	S	20	R	16	S	24	R	12	S	26	S	26	S	20	S	30	S	2
88	I	18	R	6	S	20	R	16	S	24	R	12	I	20	R	18	R	8	S	27	S	1
89	R	6	R	6	S	18	R	6	S	28	R	10	I	20	R	6	S	20	S	27	S	1
90	S	26	S	20	S	18	R	16	S	22	S	29	S	24	S	30	S	18	S	22	S	0.5
91	R	6	R	8	I	13	R	6	I	18	R	6	S	24	R	6	S	20	S	28	S	1
92	S	26	R	8	I	13	R	18	S	30	S	26	S	30	R	6	R	6	S	24	S	1
93	S	22	S	20	S	20	R	6	S	30	S	30	S	28	R	6	S	20	S	28	S	0.5
94	R	12	R	6	I	13	R	18	I	18	R	6	R	6	R	6	R	6	S	28	S	0.5
95	R	6	R	6	R	6	R	6	R	6	R	6	S	26	S	28	S	18	S	26	S	0.5
96	R	6	R	6	R	6	R	20	R	6	R	12	S	26	R	6	R	6	S	30	S	1
97	S	22	R	10	R	6	R	20	R	6	R	12	R	12	R	14	S	20	S	30	S	1
98	S	28	S	22	S	20	R	20	S	28	R	12	S	28	R	6	S	22	S	30	S	0.3
99	R	6	R	6	R	10	R	6	I	16	R	12	S	28	R	6	S	20	S	30	S	1

100	R	6	R	6	S	18	R	20	R	6	R	8	R	10	R	21	R	6	S	30	S	0.5
101	S	22	S	20	S	18	R	20	S	26	R	6	I	16	R	6	S	20	S	28	S	0.5
102	S	26	S	20	S	20	R	20	S	26	R	6	S	26	R	6	S	22	S	28	S	0.5
103	S	22	R	6	R	8	R	22	S	24	R	12	R	12	R	18	S	20	S	26	S	1
104	S	25	S	22	S	20	R	26	S	24	S	26	S	28	S	30	S	20	S	22	S	1
105	S	24	R	6	R	6	R	24	R	6	R	12	I	16	R	6	R	6	S	26	S	0.5
106	R	6	S	18	S	18	R	24	S	24	S	26	R	6	R	6	S	22	S	24	S	0.5
107	R	6	I	12	S	18	R	24	S	24	S	26	S	26	R	6	R	6	S	32	S	1
108	S	27	R	6	R	6	R	22	R	6	R	10	R	6	R	6	S	20	S	30	S	1
109	S	22	S	18	S	18	R	22	R	6	R	10	S	28	S	30	S	22	S	30	S	0.3
110	S	22	R	6	R	6	R	22	S	26	S	30	R	12	R	6	S	20	S	30	S	1
111	R	14	S	18	S	20	S	34	S	30	R	6	S	24	R	12	S	20	S	30	S	1
112	S	28	S	1	S	20	R	22	R	8	R	6	S	24	R	6	S	20	S	28	S	1
113	S	28	R	6	R	8	R	20	R	8	R	6	R	12	R	6	S	22	S	28	S	0.5
114	S	24	R	6	S	20	R	18	R	10	R	6	I	16	S	30	S	20	S	22	S	1
115	R	6	R	6	R	8	R	6	R	6	R	12	I	16	R	6	R	10	S	28	S	0.5
116	S	22	S	24	S	18	R	6	R	6	R	12	S	28	R	6	S	22	S	24	S	0.5
117	S	26	S	20	S	18	R	6	R	14	R	12	R	6	S	32	R	10	S	28	S	0.5
118	R	6	S	18	S	18	R	6	R	6	R	10	I	20	R	6	S	20	S	30	S	1
119	S	28	S	18	S	18	R	6	S	30	R	6	S	28	R	6	S	22	S	24	S	0.5
120	S	28	S	20	S	18	R	6	S	26	R	6	S	28	S	30	S	20	S	24	S	0.3
121	S	26	S	20	S	18	S	34	R	6	R	8	R	6	S	30	S	20	S	24	S	1
122	R	6	I	12	S	20	R	6	I	16	R	8	R	12	R	6	R	6	S	30	S	1
123	S	28	R	6	R	6	R	6	R	6	R	6	R	6	R	6	R	6	S	30	S	0.5
124	S	24	S	28	R	6	R	6	R	6	R	6	S	28	S	28	S	19	S	28	S	0.5
125	R	6	S	22	S	18	R	6	S	26	R	6	R	6	R	12	R	6	S	24	S	1
126	R	12	S	28	S	18	R	6	S	26	R	12	I	16	R	6	S	20	S	32	S	0.5
127	S	22	R	6	S	18	R	6	S	30	R	12	S	28	R	6	S	19	S	22	S	1
128	S	26	S	18	S	18	R	6	R	6	R	12	S	28	R	12	R	6	S	30	S	1
129	S	26	S	18	R	6	R	6	R	6	R	12	S	26	R	6	S	19	S	24	S	1
130	R	12	I	12	I	13	R	6	R	6	R	6	S	26	R	6	R	6	S	30	S	0.3
131	S	22	R	6	I	13	R	6	S	26	R	6	S	26	S	30	R	6	S	30	S	0.5
132	S	28	S	20	S	20	R	6	I	18	S	30	S	30	R	6	S	20	S	30	S	0.3
133	S	28	R	6	R	6	R	6	R	6	R	12	I	20	R	6	R	6	S	28	S	0.3
134	S	22	S	20	S	16	R	6	S	26	R	12	R	6	R	22	I	16	S	28	S	0.3
135	R	6	S	20	S	16	S	32	S	26	R	6	S	28	R	6	S	22	S	28	S	1
136	S	24	S	18	S	18	R	6	R	6	R	6	I	12	R	6	S	22	S	30	S	0.5
137	S	22	S	18	S	18	S	34	I	18	S	28	S	26	S	32	S	24	S	24	S	0.5
138	S	28	R	6	R	6	R	6	R	14	R	10	R	6	R	6	R	6	S	24	S	0.5
139	S	28	S	18	S	18	R	6	R	6	R	12	I	16	S	28	S	20	S	32	S	0.5
140	S	22	R	6	S	20	R	6	S	26	R	12	S	26	R	6	S	20	S	32	S	1
141	S	20	S	18	S	20	R	6	S	30	R	6	S	28	S	32	R	6	S	30	S	1
142	S	26	I	12	S	20	R	6	R	6	R	6	S	28	R	6	S	22	S	32	S	0.5
143	S	26	R	8	S	18	R	6	S	27	R	6	I	16	R	6	I	16	S	22	S	0.5
144	S	28	S	20	S	18	R	6	S	30	R	13	S	24	R	6	S	20	S	28	S	1
145	S	22	S	20	S	18	R	6	S	26	R	10	S	24	R	6	S	20	S	24	S	1
146	S	22	R	6	R	6	R	6	R	8	R	12	R	6	R	6	S	22	S	32	S	0.3
147	S	26	R	6	R	6	R	6	R	8	R	12	R	6	R	6	S	20	S	32	S	0.3
148	S	26	S	18	R	8	R	6	R	6	R	12	S	28	S	30	R	6	S	32	S	0.3
149	R	6	R	6	S	16	R	6	S	24	R	8	S	28	R	14	S	20	S	36	S	1

150	S	28	R	6	R	8	R	6	R	12	R	8	I	12	R	22	S	20	S	28	S	0.5
151	R	10	S	18	S	18	R	6	R	10	R	8	S	26	R	6	R	10	S	28	S	0.5
152	S	26	S	18	S	18	R	6	S	28	R	6	S	26	S	28	S	22	S	28	S	1
153	S	22	R	6	R	10	R	6	R	6	R	6	R	6	R	6	R	6	S	28	S	0.5
154	S	27	S	18	S	22	R	6	R	6	R	6	S	26	S	28	S	20	S	28	S	0.5
155	S	24	R	6	R	10	R	6	R	6	R	6	R	12	R	6	S	20	S	28	S	0.5
156	S	24	S	20	S	20	R	6	R	6	R	6	I	16	R	6	S	21	S	28	S	0.5
157	R	8	R	8	S	20	R	6	R	10	R	12	R	6	R	6	R	6	S	26	S	0.3
158	S	26	R	8	S	20	R	6	R	10	R	12	R	6	R	14	S	19	S	22	S	0.3
159	R	6	R	6	S	20	R	6	R	6	R	12	R	6	R	6	R	6	S	28	S	0.3
160	S	24	R	6	R	12	R	6	R	6	R	12	R	10	R	6	S	20	S	24	S	0.5
161	S	24	S	20	S	24	R	6	S	28	R	12	S	27	R	6	S	22	S	28	S	0.5
162	S	22	R	6	S	18	R	6	S	28	R	12	S	30	R	6	S	20	S	30	S	1
163	S	26	S	18	S	18	R	6	S	26	R	12	S	28	S	32	S	20	S	30	S	0.5
164	S	26	S	18	S	16	R	6	S	26	R	10	R	6	R	6	R	10	S	30	S	1
165	S	26	S	18	S	20	R	6	R	6	R	6	R	6	R	6	R	10	S	28	S	1
166	R	6	S	18	S	20	R	6	R	6	R	6	S	28	R	6	R	10	S	28	S	1
167	S	22	R	6	R	6	R	6	R	14	R	6	R	12	R	6	S	22	S	32	S	0.3
168	R	6	I	12	S	20	R	6	R	12	R	6	S	26	R	6	R	6	S	30	S	1
169	R	6	I	12	I	13	R	6	S	28	R	6	S	26	S	28	S	20	S	24	S	0.3
170	S	28	S	18	S	17	R	6	S	30	S	30	S	24	S	28	S	20	S	24	S	0.3
171	R	6	S	18	S	18	R	6	S	22	R	6	S	28	R	6	I	17	S	32	S	0.3
172	S	22	S	18	S	18	R	6	R	6	R	6	I	17	R	6	R	6	S	32	S	0.3
173	S	28	R	6	S	18	R	6	S	27	R	6	S	28	S	30	S	20	S	30	S	1
174	S	28	S	20	S	18	R	6	R	12	R	6	S	28	R	6	S	22	S	32	S	0.5
175	R	14	I	12	S	20	R	6	S	29	R	6	S	25	R	6	S	20	S	24	S	0.5
176	S	22	I	12	S	20	R	6	R	6	R	6	I	16	R	6	S	20	S	32	S	1
177	S	28	S	20	S	16	R	6	S	28	R	12	S	30	S	30	S	20	S	28	S	1
178	S	28	S	20	S	22	S	34	S	28	S	26	S	26	S	28	S	22	S	28	S	1
179	S	22	S	18	R	8	R	6	S	22	R	10	I	20	R	6	S	20	S	28	S	0.5
180	S	26	S	20	R	8	R	6	R	6	R	12	R	6	R	6	S	22	S	28	S	1
181	S	26	S	18	S	20	R	6	S	30	S	32	I	16	S	26	S	20	S	28	S	0.5
182	S	26	R	6	S	20	R	6	R	8	R	6	I	16	R	22	S	20	S	28	S	0.5
183	R	6	R	6	S	16	R	6	R	6	R	6	S	29	S	32	R	8	S	26	S	0.5
184	S	24	I	12	S	16	S	34	R	12	R	10	I	16	S	36	R	6	S	22	S	1
185	S	22	R	20	S	22	R	6	S	25	S	28	R	6	R	6	S	19	S	28	S	0.5
186	S	25	S	18	S	18	R	6	R	6	R	6	R	6	R	6	R	6	S	24	S	0.5
187	R	6	R	6	R	8	R	6	R	6	R	6	R	6	R	6	R	6	S	30	S	0.5
188	R	6	I	12	S	20	R	6	R	6	R	6	S	28	S	28	S	20	S	22	S	0.3
189	I	16	R	6	S	20	R	6	R	14	R	12	S	28	R	6	S	22	S	28	S	0.3
190	S	26	S	18	I	13	R	6	S	28	S	30	R	6	R	6	S	20	S	24	S	0.3
191	R	12	R	6	R	6	R	6	R	6	R	6	R	6	R	6	S	20	S	32	S	1
192	R	6	S	20	S	17	R	6	R	6	R	6	S	26	R	6	S	20	S	28	S	0.5
193	S	26	R	6	S	17	R	6	S	26	S	26	R	6	R	6	S	22	S	28	S	1
194	S	22	S	18	R	6	R	6	S	26	S	30	S	27	R	6	S	20	S	28	S	1
195	R	6	I	12	S	20	R	6	R	6	R	10	S	27	S	30	R	6	S	28	S	1
196	S	22	S	20	S	20	R	6	S	28	S	29	I	19	R	6	R	6	S	28	S	1
197	S	22	R	8	R	6	R	6	R	8	R	6	I	16	R	6	S	22	S	28	S	0.5
198	R	6	S	18	S	18	R	6	S	26	R	6	S	30	R	6	S	20	S	26	S	0.5
199	S	20	S	18	S	18	R	6	R	8	R	6	S	26	R	18	R	10	S	22	S	1

200	R	6	R	6	R	6	R	6	R	8	R	6	R	6	R	6	R	6	S	28	S	0.3
201	S	22	S	18	S	20	R	6	R	8	R	6	R	12	R	6	S	20	S	24	S	0.5
202	S	28	S	18	S	16	R	6	S	25	R	12	S	26	R	12	S	19	S	28	S	1
203	S	26	S	18	S	16	R	6	S	22	R	12	S	28	R	6	R	6	S	24	S	0.5
204	S	26	S	18	S	16	R	6	S	30	S	30	S	26	R	6	S	22	S	32	S	0.5
205	R	6	R	6	S	16	R	6	R	6	R	10	S	26	R	6	R	6	S	30	S	0.5
206	S	22	S	20	S	20	S	33	S	28	S	28	S	26	S	28	R	6	S	30	S	0.5
207	S	20	I	12	S	20	R	6	R	6	R	6	S	30	R	6	S	19	S	28	S	0.5
208	S	20	R	6	I	13	R	6	R	6	R	6	R	6	R	21	R	12	S	28	S	1
209	R	6	R	6	S	18	R	6	S	22	R	6	S	29	R	6	S	22	S	32	S	0.3
210	S	24	S	20	S	18	R	6	R	14	R	12	R	6	R	6	S	20	S	30	S	0.3
211	S	24	S	20	S	18	R	6	S	26	R	12	S	28	S	30	R	12	S	24	S	0.3
212	S	22	S	18	S	18	R	6	R	6	R	12	R	12	R	6	S	20	S	24	S	1
213	S	22	S	18	S	18	R	6	R	6	R	12	R	12	R	6	S	19	S	32	S	1
214	S	28	I	12	R	6	R	6	R	6	R	6	I	18	R	6	R	6	S	30	S	0.5
215	S	28	S	18	R	6	R	6	S	28	R	6	I	18	R	6	S	20	S	30	S	0.5
216	S	28	S	18	R	6	R	6	S	28	R	6	S	28	R	6	S	22	S	28	S	0.5
217	S	28	R	6	R	6	R	6	R	6	R	6	R	10	R	14	S	20	S	24	S	1
218	S	22	R	6	R	8	R	6	S	26	S	29	R	6	R	6	R	6	S	32	S	0.5
219	S	22	I	12	S	20	R	6	S	26	R	10	S	32	R	6	S	20	S	28	S	0.5
220	S	22	R	6	S	20	R	6	R	6	R	10	I	18	R	22	S	19	S	28	S	0.5
221	R	6	S	20	I	13	R	6	S	28	S	28	I	16	R	6	R	10	S	32	S	2
222	S	26	S	20	S	20	R	6	R	10	R	6	S	28	S	32	S	20	S	30	S	0.5
223	S	22	S	18	R	6	R	6	S	24	R	6	S	26	R	6	R	10	S	30	S	1
