# Supplementary material for: Antimicrobial susceptibility, virulence determinants profiles and molecular characteristics of Staphylococcus epidermidis isolates in Wenzhou, eastern China
Source: BMC Microbiol. 2019 Jul 9;19:157. doi: 10.1186/s12866-019-1523-6 (PMC6617921; doi:10.1186/s12866-019-1523-6)
Supplement: Supplementary file 2 — Drug susceptibility results of 106 strains of colonized S. epidermidis”. MIC values of 10 drug susceptibility results of 106 colonized S. epidermidis. (PDF 112 kb) [file 12866_2019_1523_MOESM2_ESM.pdf]

No.	FOX		TE		SXT		CN		P		DA		E		CIP		C		LZD		VA(MIC)	
C1	S	32	S	26	S	20	S	18	S	30	S	26	S	26	S	28	S	20	S	26	S	0.25
C2	R	6	S	26	S	20	S	18	R	6	R	12	R	10	S	28	S	20	S	28	S	0.5
C3	R	6	S	22	S	18	S	16	R	6	R	6	R	6	S	24	S	22	S	26	S	0.5
C4	R	6	S	28	R	6	S	16	R	6	S	26	R	6	I	16	S	22	S	24	S	0.5
C5	S	30	S	28	S	18	S	16	S	36	R	6	R	6	S	22	S	20	S	24	S	0.25
C6	R	6	S	26	S	16	S	20	R	6	R	6	R	6	I	18	S	20	S	28	S	1
C7	S	30	S	26	S	20	S	20	S	32	S	26	S	30	S	26	S	20	S	24	S	0.5
C8	S	32	S	26	R	6	R	12	R	6	R	6	R	10	R	6	S	20	S	32	S	0.5
C9	R	12	R	6	S	20	R	6	R	6	R	6	R	10	R	6	S	20	S	29	S	0.5
C10	R	8	S	26	S	20	S	18	R	6	S	28	R	8	S	24	S	22	S	30	S	0.5
C11	S	30	S	26	S	20	S	18	R	6	S	26	S	30	S	26	S	22	S	24	S	0.25
C12	R	8	S	22	R	6	R	6	R	6	R	10	R	6	R	6	I	16	R	6	S	1
C13	R	6	S	28	S	18	S	18	R	6	S	26	S	26	S	26	S	20	S	26	S	0.5
C14	S	30	S	28	S	18	S	18	R	12	S	28	S	26	S	26	S	20	S	28	S	0.5
C15	S	32	S	26	S	16	S	16	S	30	S	28	R	6	S	26	S	26	S	24	S	0.5
C16	R	6	R	6	R	6	S	16	R	18	R	6	R	6	R	6	S	26	S	32	S	0.5
C17	R	6	S	28	S	20	S	16	R	22	R	12	S	28	S	28	S	26	S	29	S	0.5
C18	R	6	S	28	R	6	R	6	R	6	R	6	R	6	R	6	S	26	S	30	S	1
C19	R	6	R	6	R	6	R	6	R	6	R	6	R	6	I	20	S	22	S	24	S	0.25
C20	R	6	R	6	R	6	R	6	R	16	I	16	S	28	R	6	R	12	S	26	S	0.5
C21	R	14	S	26	S	20	S	18	R	16	S	32	S	28	S	26	S	20	S	30	S	0.5
C22	S	26	S	26	S	20	S	18	R	16	I	16	R	6	S	26	S	20	S	30	S	0.5
C23	R	6	S	22	S	18	S	16	R	6	R	6	R	6	R	6	S	20	S	30	S	0.25
C24	R	6	S	28	R	6	S	16	R	16	S	32	I	18	S	24	S	22	S	28	S	1
C25	R	6	S	28	R	6	S	16	S	36	S	30	R	6	S	24	S	22	S	22	S	1
C26	R	6	R	10	R	6	S	20	R	18	S	30	R	6	S	26	I	16	S	28	S	1
C27	S	30	S	28	S	20	S	18	R	6	S	28	R	12	S	26	S	20	S	28	S	0.5
C28	R	6	S	26	S	20	S	18	R	6	S	28	S	26	S	24	S	20	S	30	S	0.5
C29	R	6	R	8	S	20	S	16	R	6	S	28	R	6	S	24	S	20	S	30	S	0.5
C30	R	6	S	28	S	20	S	16	R	6	S	28	S	28	S	24	S	22	S	24	S	0.5
C31	S	28	S	30	R	8	R	6	S	16	S	26	R	6	I	16	S	22	S	26	S	1
C32	R	14	R	6	R	8	S	16	R	6	S	26	R	6	S	28	S	26	S	26	S	0.25
C33	R	6	S	26	S	20	S	18	R	6	R	6	R	10	R	6	S	26	S	22	S	0.5
C34	R	6	S	26	S	20	S	18	R	16	S	26	R	6	S	26	S	26	S	28	S	0.5
C35	R	6	S	22	S	18	S	16	R	16	R	6	R	6	S	24	S	22	S	26	S	0.5
C36	S	28	S	28	S	18	S	16	R	16	S	26	S	30	R	10	S	26	S	26	S	0.5
C37	S	30	S	28	S	18	S	16	S	34	S	28	R	6	S	26	S	26	S	32	S	0.25
C38	S	28	R	6	S	16	I	14	S	36	R	6	R	6	S	24	S	28	S	32	S	1
C39	R	6	S	26	R	8	R	6	R	6	S	28	S	28	I	16	S	28	S	30	S	0.5
C40	S	30	S	26	S	20	S	18	R	6	S	28	S	28	S	28	S	20	S	30	S	0.5
C41	S	32	S	22	S	20	S	18	S	38	S	24	R	6	S	26	S	20	S	24	S	0.5
C42	S	30	S	28	S	18	S	16	S	36	S	30	R	6	R	6	S	20	S	26	S	1
C43	S	30	S	28	S	18	S	16	S	36	S	26	S	26	S	24	S	22	S	24	S	1
C44	S	30	S	26	S	20	S	16	R	6	S	26	R	6	S	26	S	22	S	26	S	1
C45	S	30	S	26	S	20	R	6	R	6	S	24	S	28	R	6	R	6	S	28	S	0.5
C46	S	26	S	26	S	18	S	20	R	6	S	30	S	28	R	6	S	26	S	24	S	0.5
C47	S	26	S	22	S	18	R	10	R	6	S	26	S	26	S	28	S	26	S	32	S	0.5
C48	S	26	S	28	S	18	S	20	R	14	S	26	R	6	S	28	S	26	S	29	S	0.5
C49	S	26	S	28	R	6	S	18	S	36	S	24	S	28	I	20	S	26	S	30	S	0.5

C50	R	6	S	26	S	20	S	18	R	6	S	30	S	28	S	26	R	6	S	24	S	0.5
C51	R	6	S	26	S	20	S	18	R	6	S	26	S	26	S	26	S	28	S	26	S	0.5
C52	R	6	S	26	R	6	S	20	R	6	S	26	R	6	S	28	S	20	S	26	S	1
C53	R	6	R	6	S	18	S	20	R	6	S	26	S	26	S	28	S	20	S	26	S	0.25
C54	R	6	S	26	S	18	R	6	R	6	R	6	R	10	S	26	S	20	S	26	S	0.5
C55	R	14	S	26	R	6	S	18	R	6	S	26	R	10	S	26	S	22	S	26	S	0.5
C56	R	6	S	22	S	20	R	6	R	18	S	26	S	28	S	26	S	22	S	28	S	0.5
C57	R	6	S	28	S	20	R	6	R	6	S	30	S	28	S	26	S	28	S	24	S	0.5
C58	R	6	S	28	S	18	S	18	R	6	S	30	R	6	S	26	S	20	S	32	S	1
C59	R	6	R	6	S	18	S	18	R	6	S	30	R	6	R	6	S	20	S	29	S	0.25
C60	R	6	R	6	S	18	R	6	R	6	R	6	R	6	R	6	S	20	S	30	S	0.5
C61	R	6	R	6	S	20	R	6	R	18	R	6	R	30	R	6	S	22	S	24	S	0.5
C62	R	6	S	26	R	6	S	20	R	6	S	29	R	28	S	26	S	22	S	26	S	0.5
C63	S	36	S	28	R	6	S	20	R	6	S	28	R	28	S	26	S	26	S	24	S	0.25
C64	R	6	R	6	S	18	S	20	R	6	R	6	R	6	S	26	R	6	S	30	S	1
C65	S	36	S	28	R	6	S	18	R	6	R	6	R	6	R	6	S	28	S	28	S	1
C66	R	6	R	6	S	20	R	6	R	12	S	30	R	6	I	16	S	20	S	30	S	0.5
C67	S	32	S	28	S	20	S	18	S	32	I	18	S	28	S	26	S	20	S	28	S	0.5
C68	R	6	R	6	S	18	S	18	R	6	I	18	R	6	R	6	S	20	S	26	S	1
C69	S	30	S	26	S	18	S	20	S	6	S	30	R	6	S	28	S	22	S	22	S	1
C70	R	6	S	26	S	18	S	20	R	18	R	6	R	6	I	16	S	22	S	28	S	0.5
C71	R	6	S	26	S	20	S	20	R	6	S	26	S	30	S	26	S	20	S	24	S	0.5
C72	S	28	S	22	S	20	R	6	R	6	S	24	I	16	I	18	S	20	S	32	S	0.5
C73	R	6	S	28	S	20	S	18	R	6	S	30	S	26	I	18	S	20	S	30	S	0.5
C74	R	6	S	28	S	18	S	18	R	6	S	26	R	6	I	20	S	22	S	30	S	1
C75	S	28	S	26	S	18	R	10	R	6	R	6	R	6	S	24	S	22	S	24	S	0.25
C76	R	10	S	26	S	18	S	20	R	6	S	30	S	28	S	24	S	20	S	26	S	0.5
C77	R	10	S	26	S	20	S	18	R	6	S	26	S	28	R	6	S	20	S	26	S	0.5
C78	S	28	S	26	S	20	S	18	R	6	S	26	R	6	S	26	S	26	S	22	S	1
C79	R	6	S	26	R	6	S	20	R	6	R	6	R	6	R	6	R	6	S	28	S	0.5
C80	R	6	S	26	S	18	S	20	R	6	R	6	R	10	R	14	S	20	S	24	S	1
C81	S	30	S	26	S	18	S	18	R	6	R	6	R	10	S	18	S	20	S	30	S	0.5
C82	S	30	S	26	S	20	S	20	R	6	R	6	R	6	R	6	S	20	S	26	S	0.25
C83	R	14	S	22	R	6	S	20	R	14	R	6	R	6	I	18	S	22	S	26	S	0.5
C84	S	30	S	28	S	16	S	18	R	14	S	30	S	26	S	26	S	22	S	26	S	0.5
C85	S	30	S	28	S	16	S	18	S	36	S	30	S	26	S	26	S	26	S	30	S	0.5
C86	R	6	R	6	R	6	S	20	R	6	R	6	R	6	I	18	S	26	S	30	S	1
C87	R	18	S	26	I	14	S	20	R	6	S	30	R	6	S	24	S	22	S	29	S	0.5
C88	R	6	S	26	S	20	S	20	R	6	S	26	S	26	S	26	S	26	S	28	S	2
C89	R	16	S	26	S	20	S	18	R	6	S	26	S	26	R	6	S	26	S	24	S	0.5
C90	R	6	S	26	S	18	S	18	R	14	R	6	R	6	S	26	S	28	S	30	S	0.5
C91	S	32	S	22	S	18	S	20	R	20	S	26	R	6	S	26	S	28	S	30	S	1
C92	S	30	S	28	S	18	S	18	R	18	S	24	S	26	S	28	S	22	S	28	S	1
C93	S	30	S	28	S	16	S	20	S	30	S	30	R	8	S	27	S	26	S	28	S	0.5
C94	R	6	S	26	R	6	S	20	R	6	S	26	R	8	S	26	S	26	S	22	S	0.5
C95	R	6	S	30	S	20	S	18	R	6	S	26	R	8	R	6	S	26	S	28	S	0.5
C96	R	6	R	12	S	20	S	18	R	6	S	24	S	24	S	26	S	26	S	26	S	0.25
C97	S	32	R	6	R	6	S	20	R	6	S	30	S	24	S	26	R	6	S	29	S	0.5
C98	S	32	S	26	S	20	S	18	R	6	S	26	S	28	S	26	R	6	S	24	S	0.5
C99	S	30	S	26	S	20	S	20	S	36	S	26	S	26	S	26	S	24	S	24	S	0.5

C100	S	30	S	26	S	18	S	20	S	36	S	26	S	26	S	26	S	24	S	4	S	0.25
C101	R	6	R	6	S	18	S	18	R	6	S	26	S	26	S	26	S	24	S	24	S	1
C102	S	32	S	26	S	18	S	18	R	6	S	26	S	26	S	26	S	26	S	32	S	0.5
C103	R	6	S	26	R	6	R	6	R	6	R	6	R	6	S	26	S	26	S	32	S	0.5
C104	R	6	S	22	S	20	S	20	R	6	R	6	R	6	S	28	R	6	S	30	S	0.5
C105	R	12	S	28	S	20	R	6	S	34	S	28	R	6	I	16	S	28	S	30	S	0.5
C106	S	30	S	28	R	6	S	20	R	12	R	6	R	6	S	26	S	22	S	28	S	0.5
